# Excimer laser electrode extraction in the presence of a leadless pacemaker: a case report

**DOI:** 10.1007/s00392-023-02229-w

**Published:** 2023-05-20

**Authors:** P. Xynogalos, N. Frey, M. Karck, R. DeSimone

**Affiliations:** 1 Department of Cardiology, University Hospital Heidelberg, Heidelberg, Germany; 2 Department of Cardiac Surgery, University Hospital Heidelberg, Heidelberg, Germany

Sirs,

The incidence of electrode dysfunction is rising worldwide, since the indication for cardiac electronic implantable devices has been broadened and the population is ageing [6]. Furthermore, the haemodynamic effects of transvenous leads causing tricuspid valve regurgitation have recently been in focus [1]. Lead extractions are increasingly necessary to provide solutions for patients with electrode failure, infection or tricuspid valve regurgitation. However, leads with longer implant duration and strong adhesions pose a risk factor for procedural failure and complications. The laser extraction system is an established extraction solution with low risk and high success rates [7].

Newer systems such as leadless pacemakers are a valuable addition to the arsenal of the electrophysiologist, since they avoid pocket-related complications as well as tricuspid valve dysfunction [5]. However, a lead extraction with a leadless pacer present includes a higher risk for dislodgement, as the right ventricle can be massively deformed during the extraction manoeuvres. We report here a case of successful lead extraction with the use of excimer laser in the presence of a leadless pacemaker.

An 80-year-old patient was referred to our centre for evaluation of the therapeutic strategy. The patient had his first transvenous pacemaker in the year 2002 and subsequently underwent a change of the battery in the year 2012. Now, he presents with pacemaker dependence, a beginning dysfunction of the old transvenous electrode with impedance changes up to 1500 Ω and a significant tricuspid valve regurgitation. The patient has a post-capillary pulmonary hypertension caused by diastolic left ventricular dysfunction, with dilatation of the right ventricle and tricuspid valve annulus and good right ventricular function. However, the transoesophageal echocardiography revealed that the pacemaker lead obstructed the movement of the tricuspid valve leaflet, thus contributing to severe tricuspid valve regurgitation (Fig. 1A). An epimyocardial pacemaker implantation was not desired by the patient as he had concerns about MRI conditionality. Therefore, an individual strategy was chosen: we implanted a leadless pacing system (MICRA Medtronic Corporation) in a septal position (Fig. 2A). After 8 weeks, having assured good measurements of the MICRA system (and possibly a partial encapsulation), we performed a transvenous lead extraction using an excimer laser (Excimer Laser System OP-Laser CVX-300, Philips Corporation). Despite concerns regarding right ventricular deformation during extraction and subsequent dislodgement of the leadless pacing device, the extraction was performed without any occurrences. The measurements before and after the extraction were identical and the radiologic imaging showed no change in the leadless pacemaker (Fig. 2B). The echocardiography after extraction demonstrated a reduction of the tricuspid valve regurgitation, thus conferring a possible haemodynamic benefit for the patient (Fig. 1B). Tricuspid valve regurgitant volume measured with the PISA method showed an initial regurgitant volume of 98.28 ml, which was reduced to 39.53 ml after the explanation.

**Fig. 1:**
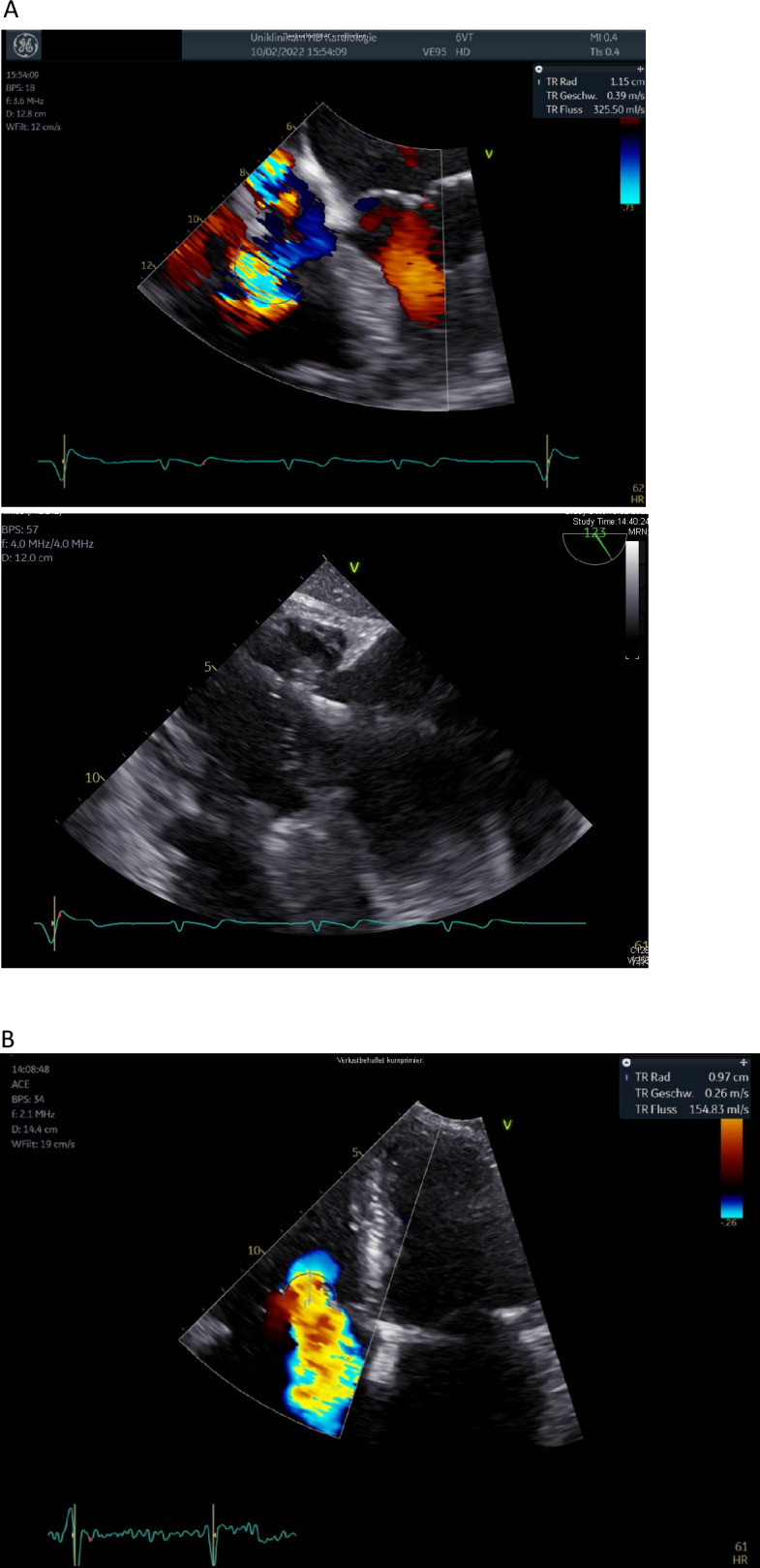
**A** Transoesophageal echocardiography before lead extraction. Shown here is the mechanical contribution of the electrode to the regurgitation by hindering leaflet closure as well as the Doppler signal of the regurgitation (PISA radius 1.53). **B** Echocardiography after lead extraction demonstrating a significant reduction of tricuspid regurgitation after lead extraction (PISA radius 0.97)

**Fig. 2:**
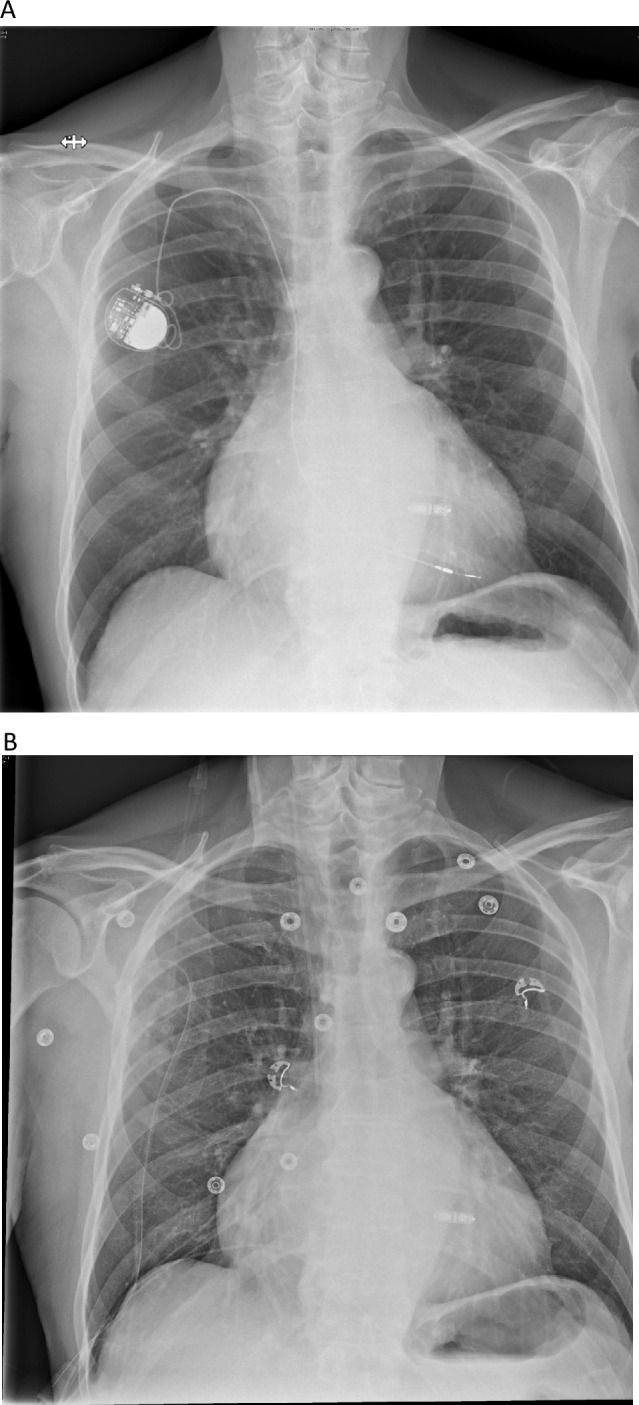
**A** Chest X-ray after MICRA Implantation and prior to lead extraction. **B** Chest X-ray after lead extraction showing stable position and no dislocation of the leadless system

Despite the fact that the absence of transvalvular electrodes could contribute to the betterment of tricuspid valve regurgitation, leadless pacing may still affect tricuspid valve function negatively. Detailed measurements of tricuspid valve regurgitation as well as long-term follow-up data for this patient are not available. Prognostic relevant echocardiographic parameters of the RV function are not available. Therefore, the prognostic benefit of this strategy regarding RV failure remains undefined.

This is to our knowledge the first report of laser-assisted electrode extraction of a 20-year-old lead in the presence of a leadless pacing system. Concerns regarding the risk of dislodgement of the leadless pacing system during extraction of the transvenous electrode should be considered. Here, the use of laser assistance could be of help, as mechanical stress is less compared to other mechanical extraction methods.

To date, published cases of electrode extraction and leadless pacemaker implantation utilized the strategy of first extracting the electrodes (with temporary pacemaker implantation where needed) and consequently leadless pacemaker implantation, possibly due to concerns of dislocation when extracting after implantation of a leadless pacemaker [2, 4, 8]. In the published data from Bicong et al. [3] only 1 of 39 patient with CIED infection underwent leadless pacemaker implantation the day before complete hardware removal. In this case, the use of an extraction system has not been reported.

As cardiac electronic implantable device failures of old systems and venous access problems by stenosis or occlusion are a clinical reality, the option of implanting a leadless pacing system is a viable solution. As a consequence, electrophysiologists and heart surgeons will be confronted with the management of old inactive electrodes in the presence of active leadless systems. A laser-assisted extraction could be considered in such cases.

## Data Availability

Not applicable.
